# Automatic detection and segmentation of adenomatous colorectal polyps during colonoscopy using Mask R-CNN

**DOI:** 10.1515/biol-2020-0055

**Published:** 2020-08-14

**Authors:** Jie Meng, Linyan Xue, Ying Chang, Jianguang Zhang, Shilong Chang, Kun Liu, Shuang Liu, Bangmao Wang, Kun Yang

**Affiliations:** Department of Gastroenterology and Hepatology, Tianjin Medical University General Hospital, Tianjin 300052, China; Department of Gastroenterology, Affiliated Hospital of Hebei University, Baoding 071000, China; College of Quality and Technical Supervision, Hebei University, Baoding 071002, China

**Keywords:** CRC, adenomatous polyps, CAD, CNN, colonoscopy

## Abstract

Colorectal cancer (CRC) is one of the main alimentary tract system malignancies affecting people worldwide. Adenomatous polyps are precursors of CRC, and therefore, preventing the development of these lesions may also prevent subsequent malignancy. However, the adenoma detection rate (ADR), a measure of the ability of a colonoscopist to identify and remove precancerous colorectal polyps, varies significantly among endoscopists. Here, we attempt to use a convolutional neural network (CNN) to generate a unique computer-aided diagnosis (CAD) system by exploring in detail the multiple-scale performance of deep neural networks. We applied this system to 3,375 hand-labeled images from the screening colonoscopies of 1,197 patients; of whom, 3,045 were assigned to the training dataset and 330 to the testing dataset. The images were diagnosed simply as either an adenomatous or non-adenomatous polyp. When applied to the testing dataset, our CNN-CAD system achieved a mean average precision of 89.5%. We conclude that the proposed framework could increase the ADR and decrease the incidence of interval CRCs, although further validation through large multicenter trials is required.

## 1 Introduction

Colorectal cancer (CRC), also known as colon or bowel cancer, ranks at top 2 and 3 of the most commonly diagnosed cancers and high mortality cancers worldwide, respectively, including an estimated 1.8 million cases with 881,000 deaths reported in 2018 [1]. Previous clinical observational studies revealed multiple stages of CRC development [2] and found that most malignancies originated as adenomatous polyps [3,4]. Therefore, CRC prevention efforts have been directed at the early detection and subsequent removal of adenomatous polyps [5,6,7].

Various screening techniques are available for CRC detection. Of these, colonoscopy has become the most widely used [8] and is recommended as the test of choice in the American College of Gastroenterology CRC Screening Guidelines [9]. The primary indicator of colonoscopy quality is the colonoscopist’s adenoma detection rate (ADR), which reflects the ability to identify adenomas. The risk of post-colonoscopy CRC is correlated negatively with an endoscopist’s ADR, as demonstrated in a large Kaiser Permanente study wherein the risk of interval CRC decreased by 3–6% with every 1% increase in ADR [10]. The ADR is affected by both colonoscopist- and procedure-dependent factors, including the training level, cecal intubation rate, withdrawal time and bowel preparation quality [11]. Unfortunately, the actual use of ADR varies widely among colonoscopists who perform the screening procedures, with reported rates varying from 7 to 53% [12].

Computer-aided diagnosis (CAD) is a computer-based technology developed to reduce and streamline the workloads of clinicians and has been applied to breast cancer [13,14], brain tumors [15,16] and pulmonary nodules [17,18]. Moreover, artificial intelligence technology could potentially enable the automated detection of polyps during colonoscopy. Karkanis et al. [19] first proposed a CAD model for the detection of adenomatous polyps based on color and texture analyses. Subsequent studies further evaluated the usefulness of shape [20,21], spatio-temporal [22] and edge features [23]. However, these algorithms were based mainly on traditional machine learning techniques, which rely heavily on image preprocessing and subsequent feature extraction by human programmers.

Deep learning is a data-driven machine learning method and has markedly advanced the computer vision to the state-of-the-art. Compared with traditional machine learning strategies, the advantage of deep learning involves the capacity to learn features automatically from large training sets without informing the computer about unique features. Consequently, multiple frameworks that adopt deep convolutional neural networks (CNNs) have been used in polyp detection applications. For example, Li et al. [24] described the use of a deep learning model for polyp detection, although their system only achieved a less than ideal accuracy (86%) and sensitivity (73%). Byrne et al. [25] applied deep learning to the real-time recognition of neoplastic polyps from a colonoscopy video and achieved an accuracy of 83%. Zhang et al. [26] proposed a strategy for the transfer learning of CNN features from a non-medical to a medical domain and trained a standard support vector machine (SVM) classifier to perform polyp detection and classification. Using fully connected convolution networks, Brandao et al. [27] detected polyps in a publicly available colonoscopy dataset and achieved an intersection over union (IoU) of 47.78%. Although the above-listed algorithms can detect adenomatous colorectal polyps, they are hindered by the following major shortcomings. First, the performances were overfitting because the proposed methods were tuned to obtain the best possible accurate detection results for the corresponding publicly available datasets. Second, the sample sizes were small and did not enable the use of separate training and testing sets of essential labeled medical images. Third, these studies paid little attention to the accurate and objective diagnosis of adenoma which, as we mentioned above, is more important for CRC prevention.

In this study, we apply an improved algorithm based on deep neural networks to achieve the segmentation of specific adenomatous polyps on RGB images obtained via conventional white-light endoscopy. Our model can automatically extract multiple-scale features from lots of colonoscopy images, as well as construct hierarchical residual-like connections within one single residual block to improve the multiple-scale representation ability at a more granular level. The major contributions of this work are as follows: (1) our CAD-CNN system can extract automatically the colonoscopy image features, rather than extracting manually with extensive preprocessing; (2) we improved the Mask R-CNN [28] (particularly the backbone structure) by constructing hierarchical residual-like connections within one single residual block to improve the multiple-scale representation ability at a more granular level; (3) the proposed system is able to detect and segment adenomatous polyps from colonoscopy images and address the polyp detection task with a variable scale.

Using our model, we pretrained Mask R-CNN on a COCO dataset and appropriately fine-tuned the algorithm using manually labeled images from the videos of actual endoscopic colonoscopies performed at a single center. We then assessed the usefulness of our method on an adenoma dataset that was split into a training and a testing set. We demonstrated that our method achieved a better result than other state-of-the-art detection methods, with a mean average precision (mAP) of 89.5%.

## 2 Materials and method

### 2.1 Dataset

For our analysis, we collected a total of 50,230 colonoscopy images from the reported 1,197 patients who underwent colonoscopy examinations at the Affiliated Hospital of Hebei University, China, between June 2016 and March 2019. All colonoscopy reports described the detection of at least one adenomatous polyp that was confirmed by histology. All colonoscopy images containing adenomatous polyps were correctly labeled by colonoscopists. The set of 50,230 colonoscopy images contained 2,128 (4.24%) images of unique polyps of all sizes and morphologies and 48,120 (95.76%) images without polyps. We selected 3,375 hand-labeled images from the datasets and divided them into two sets. (1) The training set comprised 3,045 images, including 1,900 with polyps (62.43%) and 1,145 without polyps (37.57%). These images were used to optimize the network parameters. (2) The testing set comprised 330 images, including 228 with polyps (69.09%) and 102 without polyps (30.91%). This set was used to estimate the actual learning ability of the network and determine the potential overfitting of the model on the training data.

### 2.2 Training architecture and framework

There have been significant advances in extensive computer vision tasks with state-of-the-art performance followed by various deep-learning methods based on CNNs (ConvNets) [29]. While it has been shown that depth increase would lead to performance improvement, the state-of-the-art deep learning models beyond 50 layers could not take advantage of this increase due to the vanishing gradient problem [30,31,32]. Therein, the models underwent performance degradation beyond a moderate number of layers. Subsequently, residual architecture (ResNet) [32] introduces short connections to neural networks, and thereby it was proposed to solve the vanishing gradient problem while obtaining much deeper network structures.

Multiple-scale feature methods have been widely applied not only in conventional feature design but also in deep learning. To obtain multiple-scale representations, feature extractors with a wide range of receptive fields are used. CNNs instinctively learn and get coarse-to-fine multiple-scale features with a series of convolutional operators. Because of such an inherent multiple-scale feature extraction ability, CNNs can effectively solve extensive computer vision tasks. Due to the short connections to neural networks, ResNet has a large number of equivalent feature scales during the feature extraction procedure. To verify the reliability of the architecture of our choice, we performed a contrast experiment with some excellent deep learning algorithms, such as Mask R-CNN, Unet, DeepLabV3 and FPN. The experimental comparison results are shown in Table 1.

**Table 1:** Test results and comparison with other deep-learning architectures

Deep-learning architectures	Backbone	mAP50%	mAP70%	mAP75%
Mask R-CNN	ResNet-50	86.89	78.44	76.85
	ResNet-101	87.98	77.30	72.18
	**Res2Net-50**	**88.30**	**79.80**	**78.10**
	**Res2Net-101**	**89.50**	**78.40**	**73.50**
	VGG16	87.50	83.40	82.00
	SE-ResNet50	87.90	81.30	79.60
U-Net	ResNet-50	70.40	67.70	66.00
	ResNet-101	76.50	66.30	64.90
	Res2Net-50	71.60	68.85	67.12
	Res2Net-101	77.72	67.36	65.94
	VGG16	70.50	67.20	66.10
	SE-ResNet50	72.90	71.30	70.20
DeepLabV3	ResNet-50	68.20	65.90	64.50
	ResNet-101	67.80	65.10	63.60
	Res2Net-50	69.43	67.09	65.66
	Res2Net-101	69.70	66.08	64.55
	VGG16	68.90	65.67	64.60
	SE-ResNet50	69.30	65.60	63.90
FPN	ResNet-50	65.70	64.10	62.70
	ResNet-101	70.30	66.80	65.10
	Res2Net-50	66.82	65.19	63.77
	Res2Net-101	71.35	67.80	66.08
	VGG16	66.50	61.80	59.30
	SE-ResNet50	66.10	61.30	59.10

### 2.3 Functional architecture of our algorithm

A CNN is a type of feedforward neural network wherein an artificial neuron can respond to the surrounding units and process large-scale images. The CNN is a multi-layer perceptron that contains a convolution layer and a pooling layer and is inspired by the process of biological investigation. The operational methods and functions of each category and level in a CNN are distinct. The CNN has become a representative deep learning algorithm and a state-of-the-art method used for image segmentation protocols, including object recognition, object detection, semantic segmentation and instance segmentation.

In our study, we constructed an automatic polyp detection system based on Mask R-CNN, a superior performing general framework in the field of instance segmentation. Mask R-CNN is able to realize object detection in an image and segmentation mask generation toward each event simultaneously. This framework extends the Faster R-CNN, which consists of two modules: a deep, fully convolutional network that generates region proposals and a Fast R-CNN detector that uses these proposed regions. The coordination of the whole system is only for object detection. Unlike Faster R-CNN, Mask R-CNN includes a branch used to predict the mask of an object in parallel with the existing branch for object recognition with a bounding-box. This framework can be trained easily, and it runs at a speed of 5 fps, thus increasing the overhead of Faster R-CNN only slightly.

**Figure 1:**
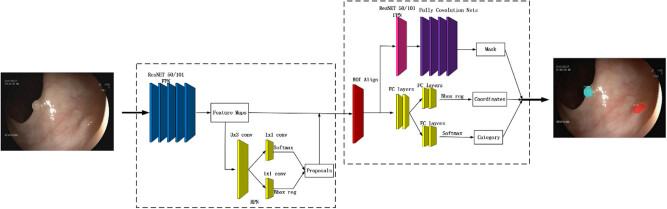
The functional architecture of Mask R-CNN.

Figure 1 illustrates the functional architecture of Mask R-CNN in our algorithm. Note that we have applied a two-stage Mask R-CNN procedure, respectively, shown in the left and right dashed boxes. The first stage aims to propose candidate bounding boxes of objects, and the second one aims to predict the class and box offset as well as a binary mask for each region of interest (RoI).

### 2.4 Improved bottleneck structure

The first stage of our algorithm based on Mask R-CNN mainly includes extracting features and proposing candidate object regions. Extraction of features over an entire image is obtained by the backbone CNNs, which extract feature maps from the input images with convolution. So, designing a more efficient network architecture is important to improve the performance of CNNs. Advances in the convolutional backbone architecture are able to enhance the ability of multiple-scale representation.

**Figure 2:**
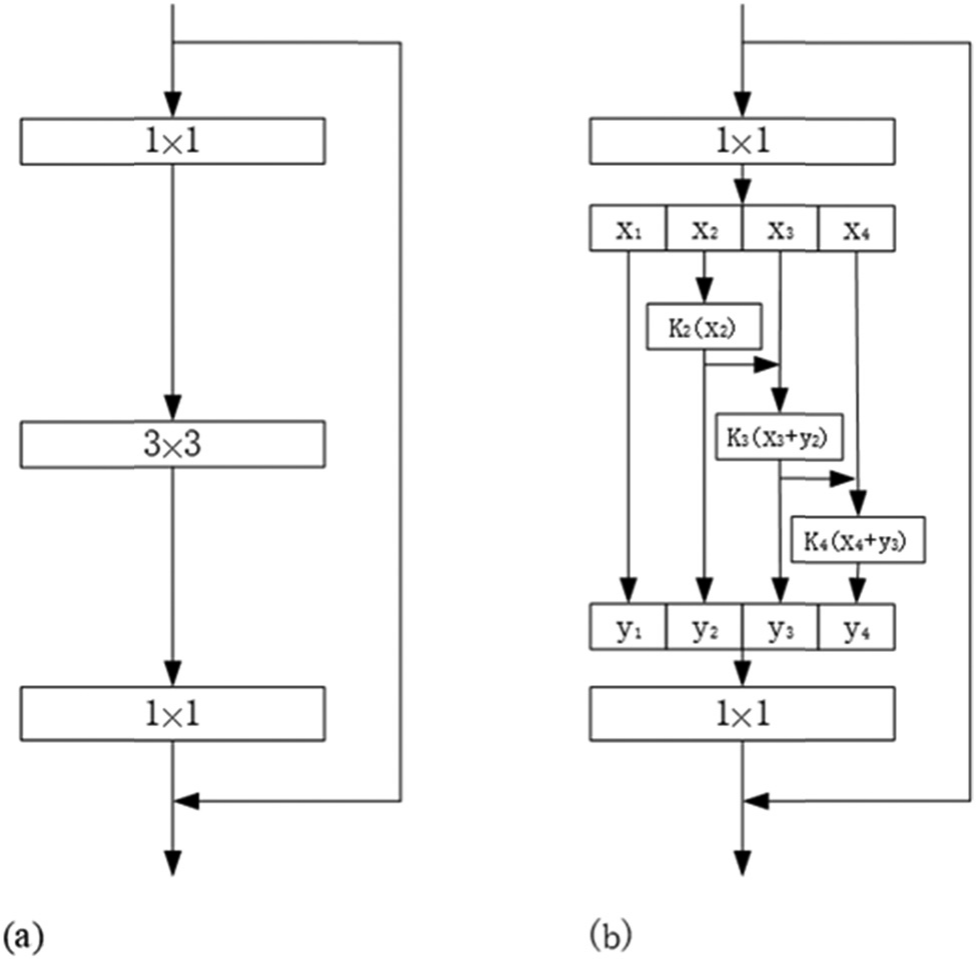
(a) The bottleneck block and (b) the Res2Net module.

As a basic building block, the bottleneck blocks extract features from the colonoscopy images and update the weights in training. The bottleneck block employed in the main-network is demonstrated in Figure 2a. And there are many CNN architectures such as ResNet [32], ResNeXt [33] and DLA [34] that use the bottleneck structure. The block includes three convolution kernels: the first 1 × 1 convolution kernel is used to decrease the input channels in order to decrease the amount of calculations required, the 3 × 3 convolution kernel is employed to extract features for the network and then the final 1 × 1 kernel increases the channels. For our approach, we have adopted Res2Net [35], which is a modified version of ResNet as the bottleneck block. As shown in Figure 2b, instead of extracting features using a group of 3 × 3 filters as in the bottleneck block, the Res2Net uses some smaller filter groups to replace the 3 × 3 filters of *n* channels, and then a hierarchical residual-like style will connect the different filter groups. Note that *n* = *s* × *w* without loss of generality.

Note that *K*_*i*_( ) represents a 3 × 3 convolutional operator. Specifically, the Res2Net module first divides the input feature maps into *s* groups such as *x*_1_, *x*_2_,…,*x*_*s*_. Then *x*_1_ directs the output to *y*_1_, which is not involved in convolution operation to reduce calculation. But for the groups *x*_2_,…,*x*_*s*_, features will be extracted using *K*(*i*_2_ ≤ *i* ≤ *s*). When extracting features, a few things to note are the previous group’s output features (*y*_*i*_,_2_ ≤ *i* ≤ *s*), the next group of filters (*i* + 1) and input features *x*_*i*_ + 1 (2 ≤ *i* ≤ *s*). It will take some time to realize this process. Thus, *y*_*i*_ is given by the following equation:

(1) \begin{document}
	\begin{document}{y}_{i}=\left\{\begin{array}{l}{x}_{i} \quad i=1; \\ {K}_{i}({x}_{i}) \quad i=2; \\ {K}_{i}({x}_{i}+{y}_{i-1}) \quad 2\lt i\le s.\end{array}\right.

Finally, all groups’ feature maps are connected and sent to another group of 1 × 1 filters. In the process, the input features are transformed to output features, and because the combination effects result in many equivalent feature scales, the equivalent receptive field increases whenever it passes a 3 × 3 filter.

### 2.5 Region proposal network

After obtaining the feature map of the input image from Res2net, the candidate object bounding boxes need to be proposed further. A Region Proposal Network (RPN) is first proposed in the faster region-based convolution neural network (Faster R-CNN). This algorithm proposed a network to generate the RoI, which indicates the region with fractures.

**Figure 3:**
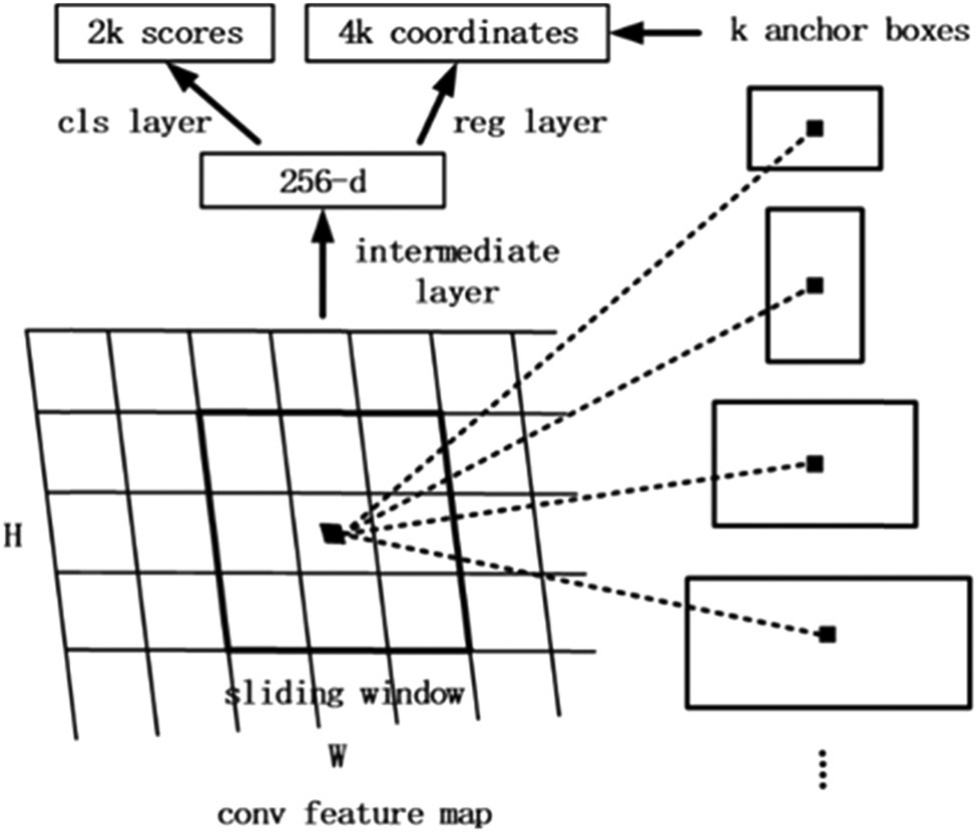
Schematic of the RPN.

Figure 3 demonstrates the procedure of the RPN. A feature map with a size of *W* × *H*, where *W* and *H* are the width and height of the feature map, is selected as the input of the region candidate network. Whereafter, by using a 3 × 3 sliding window on the feature map, an output feature map of 256 channels is generated. The size of the output feature map is identical with the input feature map, which is 256 × (*W* × *H*). It can be approximately assumed that the output feature map has *W* × *H* feature vectors, each of which has 256 dimensions. Then each feature vector is fully connected twice, one gets two scores (foreground and background) and the other gets four coordinates (*x*, *y*, *w* and *h*; the four coordinates represent the coordinate offset from the original image). After that, 1 × 1 convolution is performed twice for the entire feature map. We then get feature maps with the sizes of 2 × *W* × *H* and 4 × *W* × *H*, respectively. That is to say, there are *W* × *H* results, and each result contains two scores and four coordinates and maps into the original image. Every spatial pixel in the feature map corresponds to *k* boxes in the original map, so there are *W* × *H* × *k* boxes in the original map. We set the top left corner or the center of the box as an anchor. The process of region selection is actually to determine whether these boxes are objects and their offsets. By creating anchors on every spatial pixel in the feature map with three scales and three aspect ratios (0.5, 1 and 2), the network will generate *W* × *H* × 9 anchors. These anchors are fed to calculate the IoU with the ground-truths and assigned as a positive sample if the IoU is over its thresholds and negative otherwise.

### 2.6 RoIAlign

In the second stage, the Mask R-CNN was used to propose a RoIAlign that would remove the harsh quantization of RoIPooling by properly aligning the extracted features. This proposed change was simple because the RoIAlign layer does not quantitate the RoI boundaries or bins. The Mask R-CNN approach uses bilinear interpolation to compute the exact values of input features at four regularly sampled locations in each RoI bin. The input features can then be calculated using equations (2), (3) and (4):

(2) f({R}_{1})\approx \frac{{x}_{2}-x}{{x}_{2}-{x}_{1}}f({Q}_{11})+\frac{x-{x}_{1}}{{x}_{2}-{x}_{1}}f({Q}_{21}),\hspace{.5em}\text{where}\hspace{1em}\hspace{.5em}{R}_{1}=(x,{y}_{1}),

(3) f({R}_{2})\approx \frac{{x}_{2}-x}{{x}_{2}-{x}_{1}}f({Q}_{12})+\frac{x-{x}_{1}}{{x}_{2}-{x}_{1}}f({Q}_{22}),\hspace{.5em}\text{where}\hspace{1em}\hspace{.5em}{R}_{2}=(x,{y}_{2}),

(4) f(P)\approx \frac{{y}_{2}-y}{{y}_{2}-{y}_{1}}f({R}_{1})+\frac{y-{y}_{1}}{{y}_{2}-{y}_{1}}f({R}_{2}).

**Figure 4:**
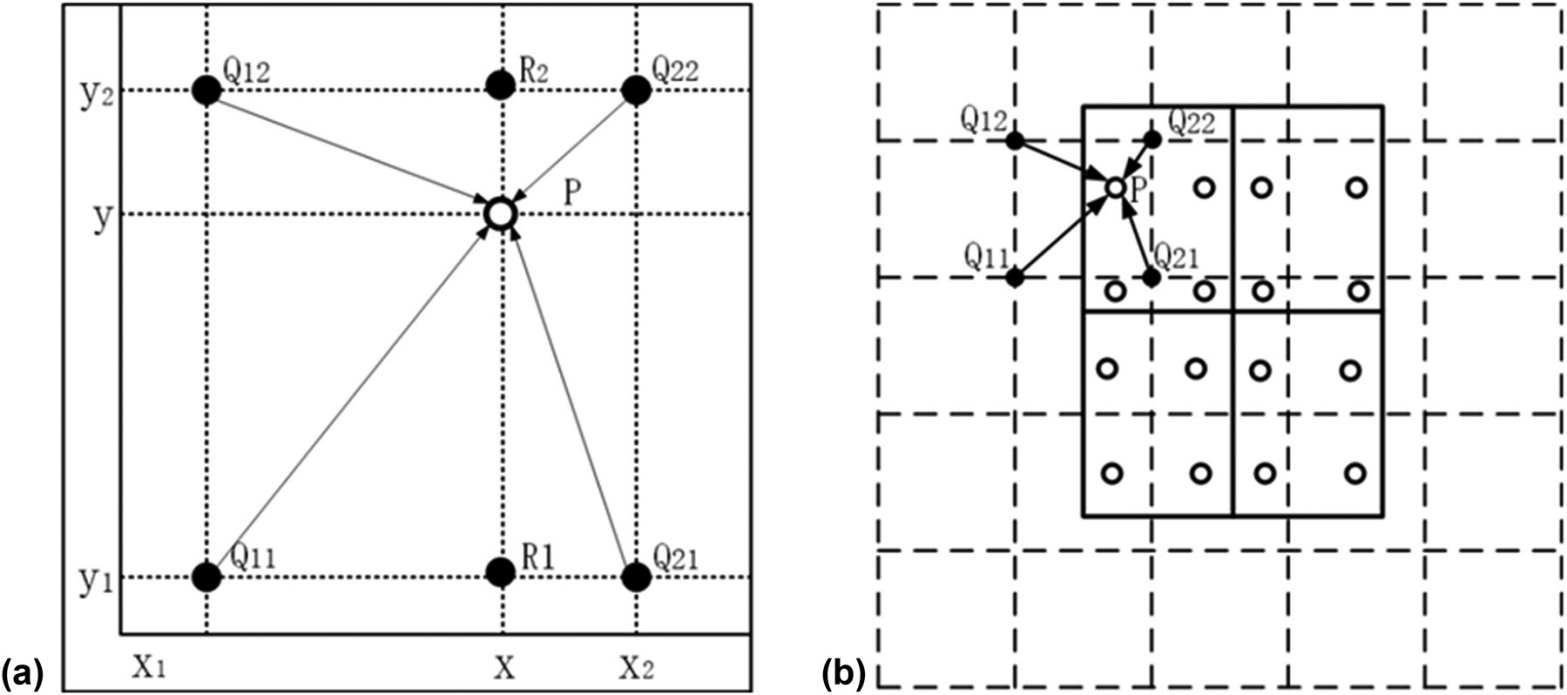
Schematic of the RoIAlign layer. (a) Bilinear interpolation and (b) the overall structure of RoIAlign.

The bilinear interpolation procedures are shown in Figure 4a. The dashed grid in Figure 4b represents a feature map. In this example, the solid lines represent an RoI with 2 × 2 bins, and the dots represent the four sampling points in each bin. RoIAlign computes the value of each sampling point via bilinear interpolation from the nearby grid points on the feature map. None of the coordinates involved in the RoI, its bins or the sampling points are subjected to quantization.

Mask R-CNN also outputs a binary mask for each RoI that parallels the predicted class and box offset. This feature differs from those of most recent systems, wherein classification depends on mask predictions. Rather, Mask R-CNN is similar to Fast R-CNN and thus applies the bounding-box classification and regression in parallel through a process that largely simplified the multi-stage pipeline of the original R-CNN.

## 3 Results and discussion

In the training process, we defined a multi-task loss as follows:

(5) \text{Loss}={L}_{\text{cls}}+{L}_{\text{box}}+{L}_{\text{mask}}.

The classification loss {L}_{\text{cls}} represents the logarithmic loss between two classes (object and non-object), and the bounding-box loss {L}_{\text{box}} is defined over a multiple of true bounding-box regression targets for class u,v=({v}_{x},{v}_{y},{v}_{w},{v}_{h}), and a predicted multiple {t}^{u}=\left({t}_{x}^{u},{t}_{y}^{u},{t}_{w}^{u},{t}_{h}^{u}\right) for class *u*. The mask branch has a *Km*2-dimensional output for each RoI, which encodes *K* binary masks of resolution *m* × *m*, one for each of the *K* classes. We then applied a per-pixel sigmoid to the Mask R-CNN and defined {L}_{\text{mask}} as the average binary cross-entropy loss. For an RoI associated with ground-truth class *k*, {L}_{\text{mask}} is only defined on the *k*-th mask on account of other mask outputs not contributing to the loss.

**Figure 5:**
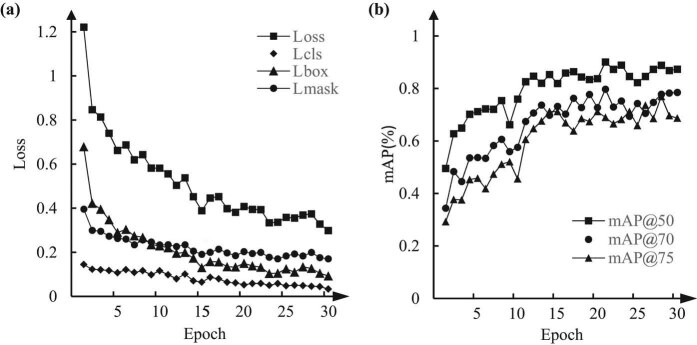
Training loss and the mAP during the epoch. (a) Training loss per iteration for a 101 layer Res2Net model on training sets. (b) Curves of the mAP during validation with the testing of training epoch.

Our deep-learning classification model with an initial learning rate of 0.001 and 0.9 learning momentum was trained using 30 epochs on the augmented training set. The values of the loss function of the training sets when training a Res2Net model with 101 layers are shown in Figure 5a. The changes in the different curves demonstrate that with increased training, the loss value decreases; moreover, the rate of this decrease becomes smaller and gradually converges to a fixed value. This phenomenon demonstrates that our Mask R-CNN-based model is a good predictor of polyps.

**Figure 6:**
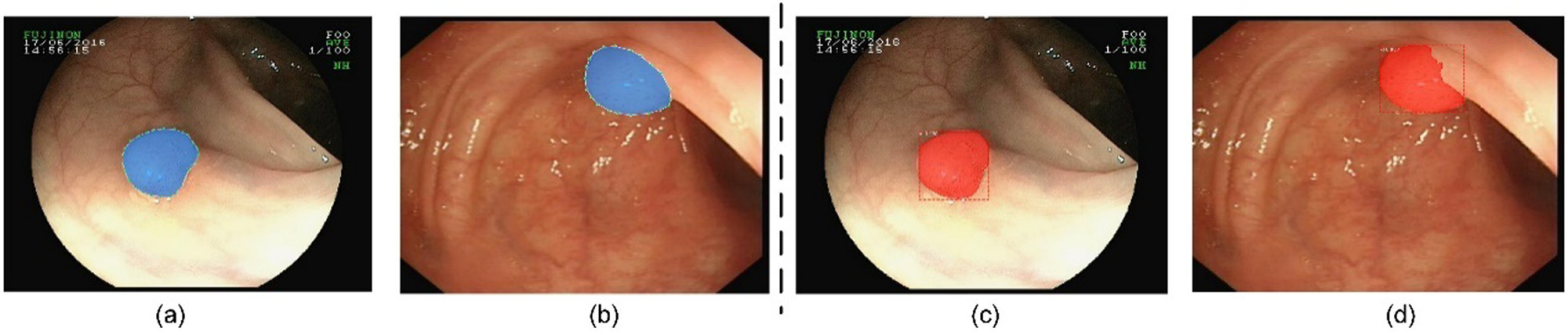
Representative images from the testing set. (a and b) The ground-truth. (c and d) The results of Mask R-CNN detection.

The results of testing are shown in two representative colonoscopy polyp images in Figure 6. In order to make sure of the accuracy of the results, we developed a unified labeling standard with the direction of the attending gastroenterologist (Figure 6a and b). Figure 6c and d presents the results of Mask R-CNN detection. Notably, in addition to detecting and providing the probability of a polyp, Mask R-CNN could also determine the location and shape of a polyp.

To further verify the performance of our polyp detection system, we generated the mAP as an evaluation metric using the following equation:

(6) \text{mAP}=\underset{0}{\overset{1}{\int }}P(R)\text{d}R,\hspace{.25em}

where *P* and *R*, respectively, denote precision and recall, as calculated in equations (7) and (8):

(7) \text{Precision}=\frac{{N}_{\text{TP}}}{{N}_{\text{TP}}+{N}_{\text{FP}}},

(8) \text{Recall}=\frac{{N}_{\text{TP}}}{{N}_{\text{TP}}+{N}_{\text{FN}}}=\frac{{N}_{\text{TP}}}{{N}_{\text{P}}}.\hspace{.25em}

Note that the various *N*s denote the numbers of true negatives, true positives, false negatives and false positives, as indicated by the subscripts.

The coincidence of the prediction frame and the real target can be clearly seen through IoU, which can directly reflect the performance of the training model. We then estimate the performance of our algorithm using three distinct IoU thresholds, 0.5, 0.7 and 0.75. As shown in Figure 5b, our method achieved mAP50, mAP70 and mAP75 of 89.5%, 78.4% and 73.5%, respectively, when applied to the testing set.

In test procedures, AP50, AP70 and AP75 in COCO style have been adopted to evaluate the results. We compared some of the state-of-the-art methods with our network on the same adenomatous polyp dataset, and Table 1 presents the results of different methods, including Mask R-CNN, U-Net, DeepLabV3 and FPN with different variations of backbone, such as ResNet, Res2Net, VGG and SE-ResNet. The boldface entries in the third and fourth rows of Table 1 show the two most accurate models using Mask R-CNN with the backbones of Res2Net-50 and Res2Net-101, respectively. It can be seen that the improved framework with the backbone of Res2Net and 101 layers is relatively higher in the average precision and reaches 89.5% AP50, which outperforms other methods. The superior performance of the Res2Net deep-neural network architecture with 101 layers was confirmed by our ablation test results in our classification work.

## 4 Conclusion

In this work, we proposed and explored an automated CNN-based CAD system based on state-of-the-art, deep-neural network architecture for CRC diagnosis. This system was used to identify adenomatous colorectal polyps as reflected in colonoscopy images. We evaluated our system with 330 images for the trial detection of colorectal adenoma as outlined by the US multi-society task force guidelines for CRC risk assessment and surveillance. The performance of the proposed method, when applied to the testing data set, was evaluated using the mAP at multiple IoU values which yielded good results. The results show that deep learning methods are available in CAD systems for the detection and diagnosis of colorectal polyps. Our experiments have received the approval of colonoscopists at the Affiliated Hospital of Hebei University. Despite the good results, larger multicenter trials are needed further to validate the ability of our proposed framework, to increase the ADR and reduce the incidence of interval CRC. Given the current complementary nature of a good classification algorithm and high-quality data, we aim to verify the system further by exploring a better backbone structure and collect a larger set of higher-quality colorectal polyp data in the future.
